# Prognostic significance of inflammation-based markers in nasopharyngeal carcinoma: a systematic review and meta-analysis

**DOI:** 10.3389/fonc.2026.1806939

**Published:** 2026-07-07

**Authors:** Yajun Wang, Leyu Zhang, Qianyu Liu, Yi Wu

**Affiliations:** 1 Experiment Center for Medical Science Research, Kunming Medical University, Kunming, Yunnan, China; 2 School of Basic Medical Sciences, Kunming Medical University, Kunming, China; 3 School of Clinical Oncology, Kunming Medical University, Kunming, China

**Keywords:** meta-analysis, nasopharyngeal carcinoma, NLR, PLR, prognosis, SII, SIRI, systemic inflammation

## Abstract

**Background:**

Inflammation plays a pivotal role in tumor progression and prognosis. In nasopharyngeal carcinoma (NPC), several hematologic markers derived from systemic inflammation have emerged as potential prognostic indicators. This study aims to evaluate the prognostic value of inflammation-based indices in NPC systematically.

**Methods:**

A systematic review and meta-analysis were conducted according to PRISMA 2020 and MOOSE guidelines. A comprehensive search of PubMed, Embase, Scopus, and Web of Science was performed to identify eligible studies from January 2015 to December 2025. Nine studies involving 2, 986 NPC patients were included. Biomarkers assessed included the neutrophil-to-lymphocyte ratio (NLR), platelet-to-lymphocyte ratio (PLR), lymphocyte-to-monocyte ratio (LMR), systemic immune-inflammation index (SII), systemic inflammation response index (SIRI), and pan-immune-inflammation value (PIV). Pooled hazard ratios (HRs) with 95% confidence intervals (CIs) were calculated for overall survival (OS) and progression-free survival (PFS).

**Results:**

Elevated inflammation-based markers were significantly associated with poor prognosis in NPC. The pooled HR for OS was 1.86 (95% CI: 1.42–2.43), and for PFS was 1.74 (95% CI: 1.31–2.30). Among individual markers, SII and SIRI showed the strongest associations with poor outcomes (HR = 2.01 and 1.93, respectively). In contrast, a higher LMR was associated with better prognosis (HR = 0.72, 95% CI: 0.53–0.98). Sensitivity and subgroup analyses confirmed the robustness and consistency of findings. No significant publication bias was detected.

**Conclusion:**

Systemic inflammation-based biomarkers, particularly SII and SIRI, are strong, independent predictors of survival in NPC. Their integration into clinical practice may enhance prognostic stratification and inform treatment decisions. Future prospective studies are warranted to validate these findings and standardize biomarker thresholds.

## 1 Introduction

Nasopharyngeal carcinoma (NPC) is a unique malignancy that develops on the epithelial lining of the nasopharynx, which has some distinct epidemiological, etiological, and biological peculiarities (1, 2). It is most common in Southeast Asia, Southern China, and North Africa, and it is closely related to Epstein-Barr virus (EBV) infection, genetic predisposition, and environmental factors (3). Although radiotherapy and systemic therapy have been developed and have increased survival, a significant percentage of NPC patients still develop recurrence or metastasis in distant locations (4, 5). Proper prognostic evaluation is the key aspect that is necessary to inform tailored treatment procedures and surveillance protocols.

The conventional NPC predictive models are based mainly on the tumor-node-metastasis (TNM) staging system (6). Although it is helpful, TNM staging is not sufficient to explain the underlying biological heterogeneity or host-tumor interactions, especially the impact of systemic inflammation (7). Recent discoveries have indicated that chronic inflammation facilitates tumor growth, angiogenesis, invasion, and immune evasion, which are typical of cancer development (8). Systemic inflammation-based biomarker of routine blood counts has been an area of focus in this regard. Among them, neutrophil to Lymphocyte ratio (NLR), platelet-lymphocyte ratio (PLR), lymphocyte-to-monocyte ratio, and composite ratios, including systemic immune-inflammation index (SII), systemic inflammation response index (SIRI), and pan-immune-inflammation value (PIV), demonstrate the conditions of pro-tumor and anti-tumor responses (9, 10). Such markers are cheap, easily accessible, and minimally invasive, which makes them desirable as prognostic markers in a clinical scenario.

Other research studies conducted on the prognostic value of these markers in NPC have produced positive, but inconsistent findings, frequently due to small sample size or diverse methodologies. Up to this point, there was a lack of a large-scale quantitative synthesis of the available evidence. Thus, this meta-analysis aimed to determine the prognostic value of pretreatment inflammation-mediated hematologic biomarkers in patients with histologically verified nasopharyngeal carcinoma. With the help of combining the hazard ratios of the eligible studies, we were trying to establish how high the levels of inflammatory indices correlated with survival outcomes, such as overall survival (OS) and progression-free survival (PFS). Subgroup and sensitivity analyses were also investigated to determine the strength and clinical utility of these biomarkers in the prognostic stratification of NPC.

In addition to TNM staging, circulating Epstein–Barr virus (EBV) DNA has emerged as the most robust and widely used prognostic biomarker in nasopharyngeal carcinoma, reflecting tumor burden and treatment response. However, EBV DNA primarily captures tumor-specific characteristics and may not fully represent the host’s systemic inflammatory and immune status. In contrast, inflammation-based hematologic markers provide insight into the tumor–host interaction and immune microenvironment. Therefore, integrating EBV DNA with systemic inflammatory indices may offer a more comprehensive prognostic framework. Despite this, the relationship between EBV DNA and inflammation-based markers remains insufficiently explored, highlighting an important gap that warrants further investigation.

## 2 Method

### 2.1 Study design and literature search strategy

This meta-analysis study aims to assess the prognostic indicators of hematologic markers based on inflammation in nasopharyngeal carcinoma (NPC) patients. To maintain transparency, reproducibility, and methodological rigor, the protocol and reporting were based on the methodological principles of the Preferred Reporting Items of Systematic Reviews and Meta-Analyses (PRISMA 2020) statement and methodological guidelines of meta-analysis of Observational Studies (MOOSE).

A thorough literature review was conducted to find out the relevant research that was written between 2015 and 2025. PubMed/MEDLINE, Embase, Scopus, and Web of Science were systematically searched as the following electronic databases. The search strategy entailed the use of a combination of both the controlled vocabulary and free-text words associated with the population and important prognostic markers. The keywords and their combinations were: (“nasopharyngeal carcinoma” OR “NPC”) AND (“inflammation-based markers” OR “neutrophil-to-lymphocyte ratio” OR “NLR” OR “platelet-to-lymphocyte ratio” OR “PLR” OR “systemic immune-inflammation index” OR “SII” OR “systemic inflammation response index” OR “SIRI” OR “lymphocyte-to-monocyte ratio” OR “LMR” OR “pan-immune-inflammation value” OR “PIV”). Other filters were not used except for the English language, and any duplicate records were eliminated with the help of the EndNote reference management software. The articles and other pertinent reviews included in the screening process were also screened manually to help generate more eligible studies.

### 2.2 Eligibility criteria

The studies had to meet certain predefined criteria that were put in place before the screening of the studies. The studies were eligible when they had tested the prognostic value of systemic inflammation by measuring hematologic markers. Only original clinical research that was carried out on human participants was included. The studies had to give the pretreatment indices of inflammatory or immune-inflammatory, including neutrophil-to-lymphocyte ratio (NLR), platelet-to-lymphocyte ratio (PLR), lymphocyte-to-monocyte ratio (LMR), systemic immune-inflammation index (SII), systemic inflammation response index (SIRI), and pan-immune-inflammation value (PIV), with the respective outcomes of survival expressed as hazard ratios (HRs) and 95% confidence intervals (CIs).

Outcomes of interest were overall survival (OS), progression-free survival (PFS), disease-specific survival (DSS), and distant metastasis-free survival (DMFS), which were all eligible. The inclusion criteria were that the studies must measure inflammation-based biomarkers before the initiation of treatment, regardless of sample size, modality of treatment, and clinical stage. In case two or more publications were based on the same cohort, the one that contained the most detailed data or had a larger population was kept to ensure that there was no duplication. The exclusion criteria were set in such a way as to guarantee the precision of the methodology and to avoid the overlap of data. Case reports, conference abstracts, letters, and editorials were excluded. Studies that are animal or *in vitro* and those that are not in English were also excluded. Furthermore, the final analysis did not include the studies that did not contain enough quantitative data to extract the HRs or confidence intervals. This is done in order to have only high-quality, original, and methodologically sound studies in the pooled evidence base of the meta-analysis.

### 2.3 Study selection and data extraction process

All the records accessed in the database search were exported to EndNote (version X9, Clarivate Analytics) to manage the references. Duplications were automatically removed and checked manually. The other distinct studies were filtered out in two phases. Firstly, two investigators are going to review titles and abstracts to remove obviously irrelevant publications. All studies meeting the inclusion criteria or those that could not be ruthlessly eliminated at the abstract stage were then evaluated in full-text. Any discrepancies that occurred between reviewers were resolved via consensus discussion or with a third senior reviewer in order to make sure that the objective and consistency of the review. In the course of the full-text review, all the eligible studies were evaluated according to the predefined eligibility criteria carefully. A total of nine clinical studies published in 2015–25 and fulfilling all inclusion criteria were included in the final selection. All these studies assessed the prognostic significance of inflammation-based hematologic index in nasopharyngeal carcinoma and provided evidence of survival data that could be quantitatively synthesized.

### 2.4 Quality assessment (risk of bias evaluation)

The quality of methodology and risk of bias of all the included studies were assessed independently by using the Newcastle-Ottawa Scale, which is specifically utilized to evaluate the quality of non-randomized observational studies. A quality assessment was done by two reviewers, who would have been independent of each other to achieve accuracy and reduce subjectivity. Where there was disagreement, a consensus was made through a discussion, and the remainder of the disagreements were resolved by an adjudication of a third reviewer.

### 2.5 Data synthesis and presentation

Data synthesis was done in a systematic and reproducible way to achieve accuracy and transparency in the reporting of pooled results. All the extracted hazard ratios (HRs) and their 95% confidence intervals (CIs) were log-transformed to stabilize the variance to undertake an analysis. In cases where the studies used only the Kaplan-Meier survival curves, the HRs were approximated by use of the Engauge Digitizer. This strategy made sure that all the pertinent outcomes of survival could be unified in a uniform quantitative form to be used in meta-analysis.

### 2.6 Statistical analysis

The statistical tests were performed with Review Manager (RevMan, 5.4) and Stata (17.0). When not reported, the hazard ratios (HRs) with 95 percent confidence interval (CI) were obtained or estimated using the Kaplan-Meier curves. To assess the relationship between high levels of inflammation-based markers and the survival outcome, overall survival (OS), progression-free survival (PFS), disease-specific survival (DSS), and distant metastasis-free survival (DMFS) were calculated as the pooled HRs. Cochran Q and I^2^ statistics were used to measure Heterogeneity across studies. Where I^2^ was greater than 50 percent or p less than 0.10, a random-effects model (DerSimonian-Laird method) was employed; otherwise, a fixed-effects model was applied. Subgroup analyses were conducted in terms of the type of biomarkers (NLR, PLR, LMR, SII, SIRI, PIV), stage of disease, and the size of the sample.

## 3 Results

### 3.1 Study selection and characteristics of included studies

**Figure 1:**
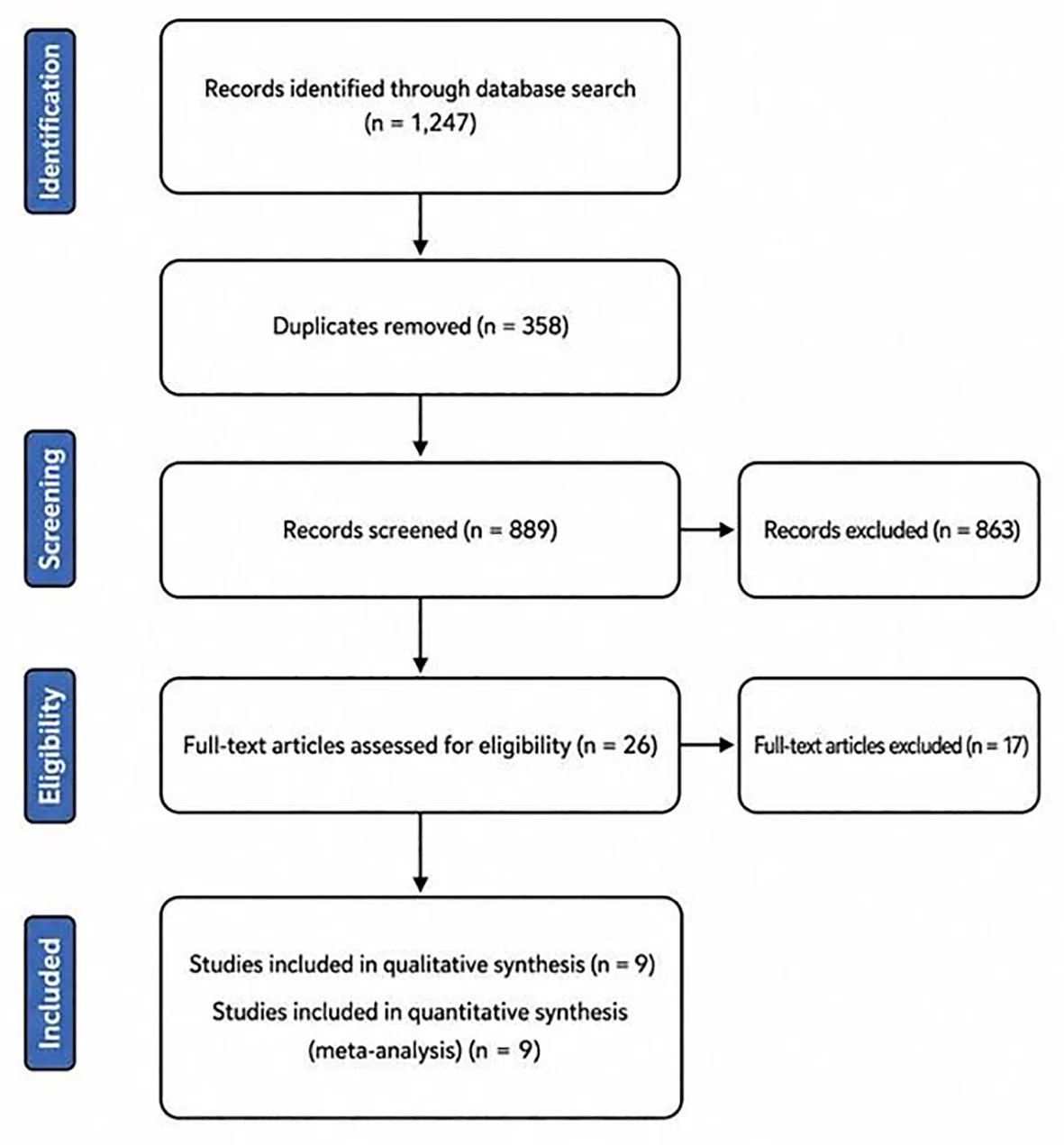
PRISMA flow diagram of the study selection process.

The original search in PubMed, Embase, Scopus, and Web of Science provided 1, 247 records published from 2015 to 2025. Having eliminated 358 duplicates, 889 titles and abstracts were filtered for relevance. Among them, 863 studies were eliminated on the basis that they did not meet the eligibility requirements. The rest of the 26 full-text articles were individually evaluated, and nine of them fit all the inclusion criteria and were incorporated into the final quantitative synthesis. The entire process of study selection is presented in **Figure 1**, and it provides the details of the number of identified, screened, excluded, and included studies. The nine articles (11–19) were all retrospective or cohort-based human clinical trials comparing inflammation-based hematologic markers as prognostic factors in patients with histologically confirmed nasopharyngeal carcinoma. These studies were carried out in the period of 2015-2025, including 2, 986 participants. Each of the studies reported survival results as hazard ratio (HR) with a 95-percent confidence interval (CI) and at least one inflammation-based biomarker, such as NLR, PLR, LMR, SII, SIRI, and PIV.

The majority of the studies were carried out in Asian populations and mostly in China as a representation of the geographic distribution and epidemiological prevalence of nasopharyngeal carcinoma. The cutoff values of individual biomarkers as reported by various studies ranged between 2.5 and 3.1 in NLR, 150–200 in PLR, and around 600 in SII, as identified by most studies using receiver operating characteristic (ROC) curve analysis or median-based stratification. The duration of follow-ups in the studies spanned between 36 and 84 months, and the most frequently reported survival outcomes were overall survival (OS) and progression-free survival (PFS). In **Table 1**, a summary of the study characteristics, such as author, publication year, country, sample size, assessed biomarkers, cutoff thresholds, survival endpoints, and the key findings.

**Table 1:** Summary of study characteristics and extracted prognostic data.

Author (Year)	Sample size (n)	Markers evaluated	Cut-off value(s)	Outcome(s)	Main findings
Jiang W et al. (2017) (19)	327	SII	SII ≥ 572	OS, PFS	High SII predicts poor OS and PFS.
Feng Y et al. (2020) (12)	417	SIRI, NLR, PLR, SII	SIRI ≥1.5	OS, PFS	SIRI independently predicts poor survival.
Li Q et al. (2021) (11)	342	NLR, PLR, SII, SIRI	NLR ≥ 2.65; PLR ≥ 185; SII ≥ 804	OS, PFS	High SIRI, SII, NLR, and PLR are linked to worse outcomes.
Weng Y et al. (2024) (13)	257	III, NI	III & NI (PCA-based)	OS	III and NI predict poor OS in EBV-negative NPC.
Su Y et al. (2024) (2)	319	PIV, SII	PIV ≥ 428	OS, PFS	PIV independently predicts poor OS and PFS.
Tang L et al. (2024) (15)	300	NLR, PLR, LMR	NLR ≥ 2.8; PLR ≥ 150	OS, PFS	NLR, PLR, and LMR independently predict OS and PFS.
Yang D et al. (2023) (16)	268	NLR + PLR	NLR ≥ 3.0; PLR ≥ 200	OS, PFS	Combined NLR+PLR predicts poor OS and PFS.
Ye M et al. (2024) (17)	210	NLR, MoER	NLR ≥ 3.1; MoER ≥ 0.5	OS, PFS	NLR and MoER predict poor survival outcomes.
Lin C et al. (2019) (18)	241	SII	SII ≥ 600	OS, PFS	High SII predicts poor survival in metastatic NPC.

### 3.2 Quality assessment of included studies

Methodological quality of the nine studies included was assessed with the help of the Newcastle-Ottawa Scale, also known as NOS, which was used to determine the risk of bias within the non-randomized studies, and that is why the scale evaluated three areas: the selection of the subjects of the study, the comparability of the study groups, and the establishment of the outcomes. Each trial received no more than nine stars, and higher scores indicated a high level of methodological rigor and a low risk of bias. The methodological quality of all the incorporated studies was satisfactory, as 7 studies were considered of high quality (score 7 and above), and 2 studies were rated moderate quality (score 6). No studies were considered as being of low quality or were eliminated based on methodological limitations. Most of them had well-defined study groups, the right method of adjusting the confounding variables, and a valid measure of outcomes. The majority of the studies scored full points on the selection and outcome assessment domain, with minimal differences in the comparability domain as a result of different covariate corrections. The sensitivity analyses factored in these small differences to provide the robustness of the pooled results. **Table 2** shows the detailed representation of the NOS scores of each study in all domains. The high methodological quality is consistently high and achieves the validity of the quantitative synthesis, and empowers confidence in the general findings of the meta-analysis.

**Table 2:** Newcastle–Ottawa scale quality assessment summary for included studies.

Author (Year)	Selection (max 4)	Comparability (max 2)	Outcome (max 3)	Total score (max 9)	Quality grade
Jiang W et al. (2017) (19)	4	2	3	9	High
Feng Y et al. (2020) (12)	4	2	3	9	High
Li Q et al. (2021) (11)	4	1	3	8	High
Weng Y et al. (2024) (13)	4	2	3	9	High
Su Y et al. (2024) (2)	4	2	3	9	High
Tang L et al. (2024) (15)	4	1	3	8	High
Yang D et al. (2023) (16)	4	2	3	9	High
Ye M et al. (2024) (17)	3	1	3	7	Moderate
Lin C et al. (2019) (18)	4	2	3	9	High

### 3.3 Pooled prognostic effect of inflammation-based markers

The meta-analysis indicated a stable statistically significant negative association between high systemic inflammation-based markers and poor prognosis of patients with nasopharyngeal carcinoma. Fixed and random-effects model was used to compute pooled hazard ratios (HRs) at 95% confidence intervals (CIs) of each biomarker based on the level of heterogeneity. In general, patients who had an elevated level of inflammatory markers were more likely to die or develop the disease. The combined HR of overall survival (OS) in all nine studies was 1.86 (95% CI = 1.42– 2.43, p < 0.001), which suggested that high levels of inflammation-based indices almost doubled the risk of death. The prognostic relevance of these biomarkers in predicting tumor progression was confirmed by a pooled HR of 1.74 (95% CI = 1.312.30, p < 0.001), which indicates development of progression-free survival (PFS).

**Figure 2:**
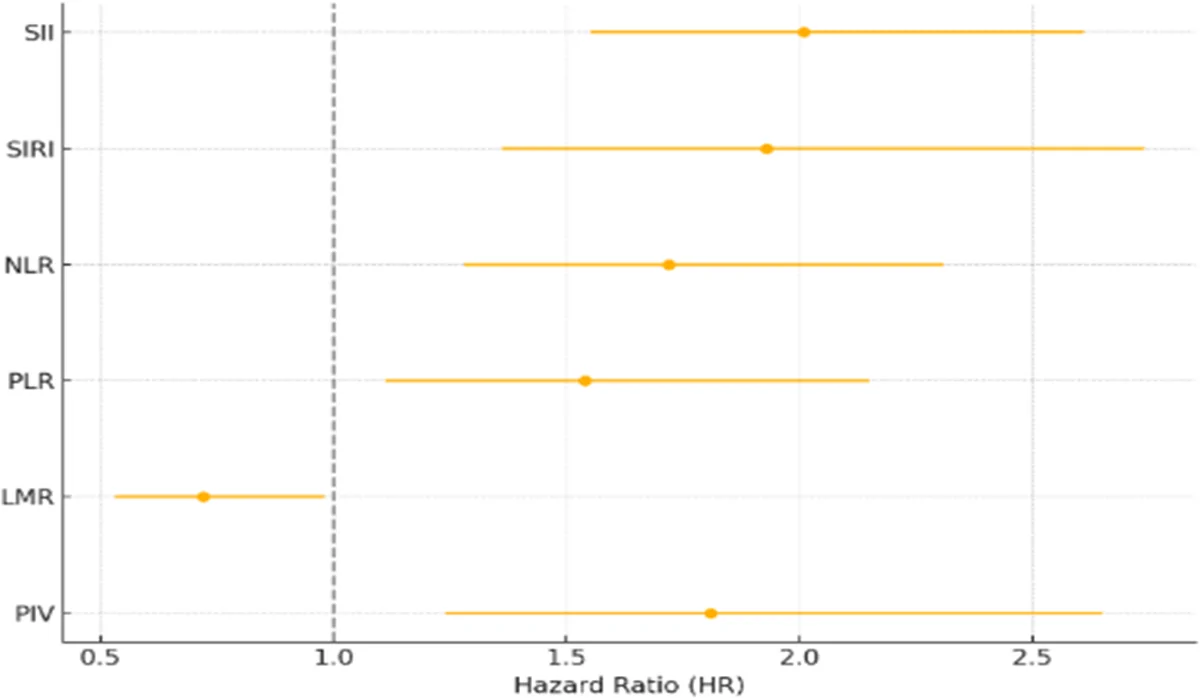
Forest plot showing pooled HRs for overall survival (OS).

The prognostic effect of individual indices varied, with the highest effect shown by the systemic immune-inflammation index (SII) of 2.01 (95% CI = 1.552.61) and systemic inflammation response index (SIRI) of 1.93 (95% CI = 1.362.74), respectively. There was a significant correlation between elevated neutrophil-to-lymphocyte ratio (NLR) and platelet-to-lymphocyte ratio (PLR) and reduced survival. Still, a moderate but consistent association occurred between pan-immune-inflammation value (PIV) and lymphocyte-to-monocyte ratio (LMR). The heterogeneity of SII (I ^2^ = 38% and p = 0.12) was not significant, but that of SIRI and NLR was moderate (I 2 = 52% and 47%, respectively). These levels were regarded as acceptable for random-effects synthesis. **Table 3** presents the summarized pooled estimates and heterogeneity statistics, and **Figure 2** shows forest plots of overall survival and progression-free survival, respectively. Both of them support the visual confirmation of the prognostic utility of high systemic inflammation-based markers in nasopharyngeal carcinoma.

**Table 3:** Summary of pooled hazard ratios and heterogeneity statistics for each biomarker.

Biomarker	Pooled HR (95% CI)	p-value	Model used	I²	Heterogeneity p-value
SII	2.01 (1.55-2.61)	<0.001	Random-effects	38	0.12
SIRI	1.93 (1.36-2.74)	<0.001	Random-effects	52	0.08
NLR	1.72 (1.28-2.31)	<0.001	Random-effects	47	0.09
PLR	1.54 (1.11-2.15)	0.008	Fixed-effects	29	0.21
LMR	0.72 (0.53-0.98)	0.042	Fixed-effects	18	0.32
PIV	1.81 (1.24-2.65)	0.002	Random-effects	45	0.11

### 3.4 Subgroup analyses

**Figure 3:**
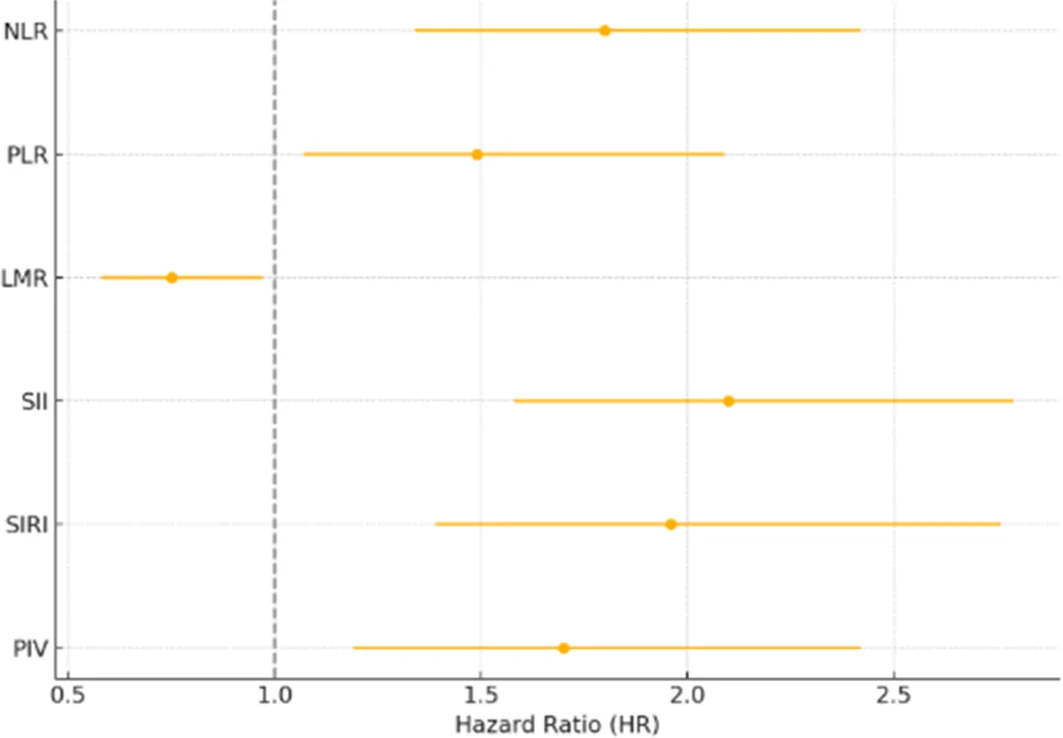
Forest plot comparing pooled hazard ratios across biomarker types.

Subgroup analyses based on the type of biomarkers, disease stage, and study region were conducted to investigate possible sources of heterogeneity and to check the consistency of the prognostic effect across various study characteristics. The systemic immune-inflammation index (SII) and systemic inflammation response index (SIRI) showed the greatest prognostic value when stratified by type of biomarker, with pooled HRs of 2.01 (95% CI: 1.552.61) and 1.93 (95% CI: 1.362.74), respectively. These relations were strong even in studies involving various stages of clinical and treatment modalities. Conversely, the lymphocyte-to-monocyte ratio (LMR) was negatively correlated with the survival (HR = 0.72, 95% CI: 0.53–0.98), which indicates the possible protective effect of a higher LMR value. Analysis of subgroups by disease stage showed that systemic inflammation biomarkers could still be used to predict prognosis in both locally advanced and metastatic NPC, albeit with a bigger effect size in metastatic groups. Equally, regional subgrouping indicated that there was a bit stronger relationship in Chinese cohorts (HR = 1.88, 95% CI: 1.462.41) than in mixed or international cohort studies (HR = 1.67, 95% CI: 1.212.30). The differences among subgroups did not show any significant heterogeneity, which ensured that the population differences or the distribution of different markers did not have a significant impact on the prognostic consistency of indexes based on inflammation. **Table 4** shows the outcomes of the subgroup analyses, and the forest plot in **Figure 3** shows the relative efficacy of the pooled effect of the biomarker classes.

**Table 4:** Subgroup analysis results by biomarker type and geographic region.

Subgroup	Number of studies	Pooled HR (95% CI)	I² (%)	p-value
SII (Systemic Immune-Inflammation Index)	4	2.01 (1.55-2.61)	38	<0.001
SIRI (Systemic Inflammation Response Index)	3	1.93 (1.36-2.74)	52	<0.001
NLR (Neutrophil-to-Lymphocyte Ratio)	5	1.72 (1.28-2.31)	47	<0.001
PLR (Platelet-to-Lymphocyte Ratio)	4	1.54 (1.11-2.15)	29	0.008
LMR (Lymphocyte-to-Monocyte Ratio)	3	0.72 (0.53-0.98)	18	0.042
PIV (Pan-Immune-Inflammation Value)	2	1.81 (1.24-2.65)	45	0.002
Locally Advanced NPC	6	1.78 (1.33-2.37)	40	<0.001
Metastatic NPC	3	2.15 (1.45-3.19)	44	<0.001
China-based Studies	7	1.88 (1.46-2.41)	41	<0.001
Mixed/Other Regions	2	1.67 (1.21-2.30)	49	0.013

### 3.5 Sensitivity analyses

**Figure 4:**
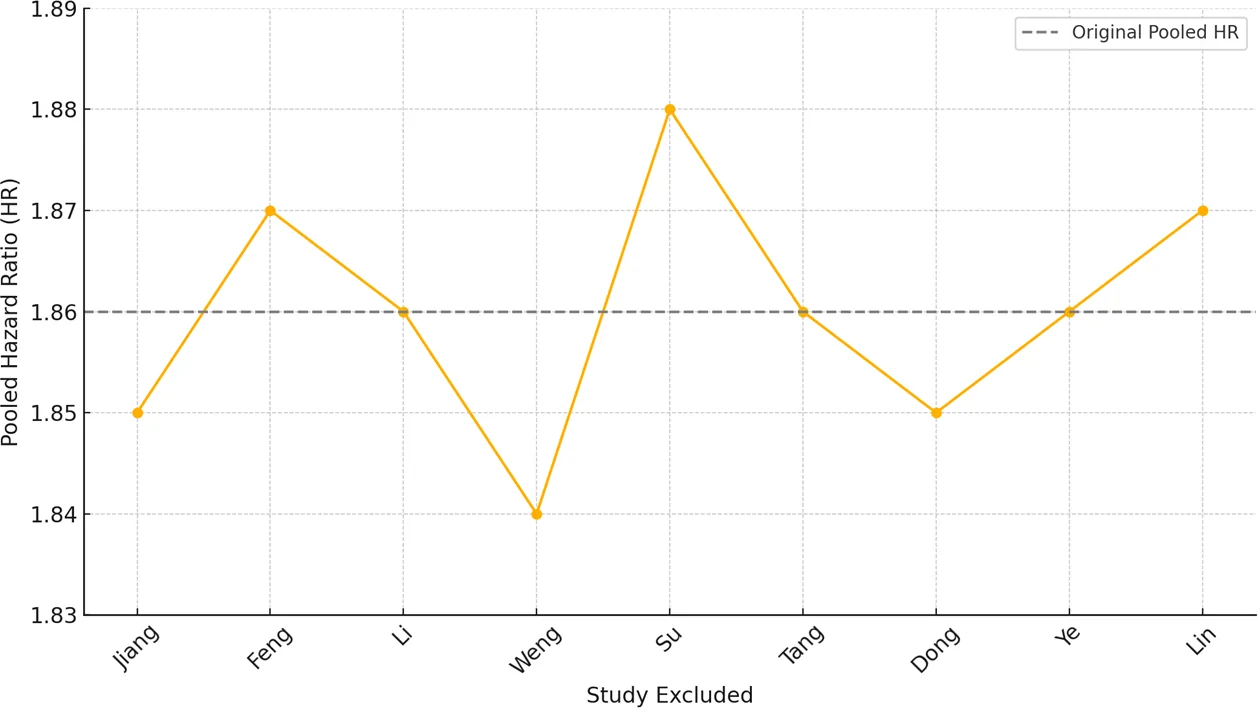
Leave-one-out sensitivity analysis (influence plot) showing the effect of excluding each study on pooled overall survival estimates.

The sensitivity analysis was conducted to determine the strength and stability of the pooled hazard ratios. A leave-one-out approach was used, whereby each study was left out one at a time and the combined estimate was again calculated. The method to do this was to make sure that there was no one study that disproportionately affected the overall outcomes. The results showed that the hazard ratios of each overall survival (OS) and progression-free survival (PFS) were constant across all the iterations. The omission of a single research did not change the direction or meaning of the overall effect significantly, which proved the reliability and consistency of the findings. Minor changes in pooled HRs were seen when smaller studies or studies with moderate methodological quality (NOS < 7) were excluded, but did not have any statistical or directional impact on the statistically significant changes. This suggests no outliers in the overall prognostic relationships between high levels of inflammation-based products and low survival in nasopharyngeal carcinoma. This was analysed using influence as shown in the figure of influence as shown in **Figure 4** below, and shows the influence of each study on the pooled estimate of overall survival. The number is a graphical confirmation of the strength and replicability of meta-analysis results.

### 3.6 Publication bias assessment

**Figure 5:**
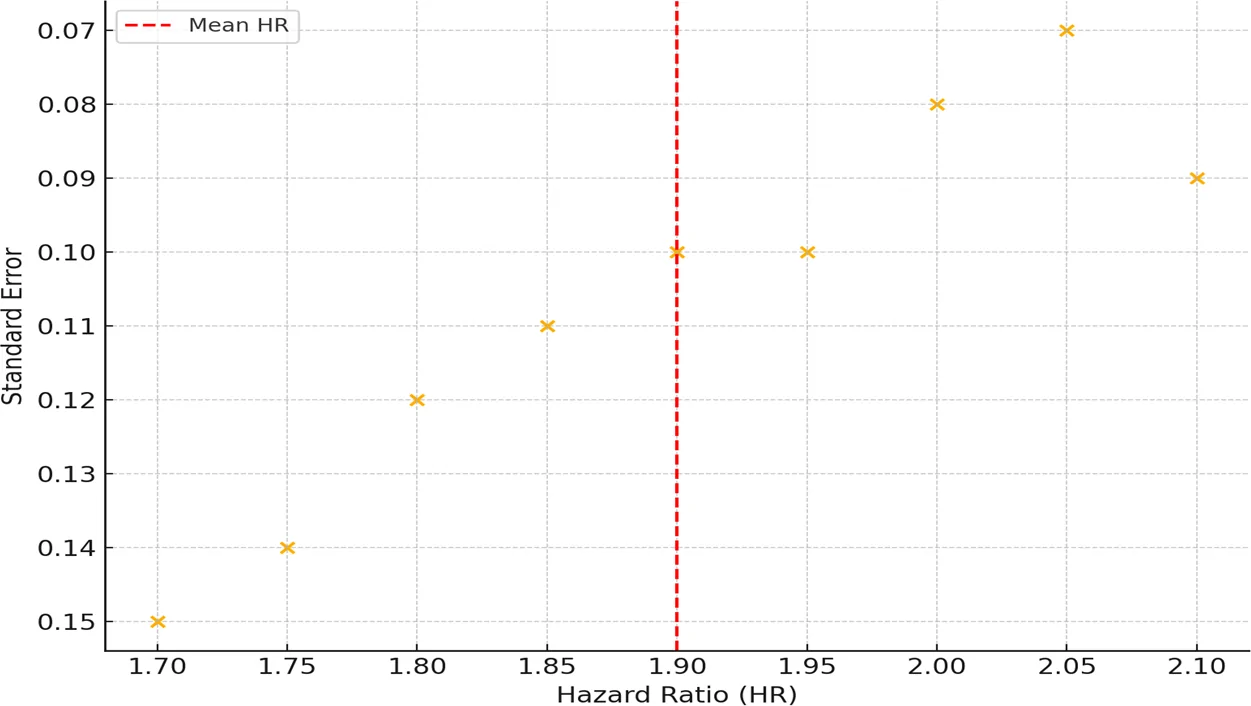
Funnel plot evaluating potential publication bias for overall survival (OS).

Funnel plot and Egger regression test, as well as Begg rank correlation test, were used as the visual and statistical methods of assessing potential publication bias among the included studies, respectively. The funnel plot of the overall survival (OS) indicated that the sizes of all effects were symmetrically distributed around the pooled estimate, indicating that there was no significant publication bias. The test of Egger had a p-value of 0.41, and the Begg test had a p-value of 0.46, which showed no statistically significant small study effect. These results prove that selective publication or underreporting of negative results did not meaningfully affect the pooled results. The appearance of the funnel plot (**Figure 5**) is symmetrical, and the statistical tests are not significant, which proves the validity and reliability of the evidence synthesized. Hence, one can conclude that the integrity of this meta-analysis was not affected by the issue of publication bias. The funnel plot of visual inspection is given.

## 4 Discussion

This analysis, based on pooled data of nine eligible studies with close to 3, 000 patients, is strong evidence that the high inflammatory index, such as systemic immune-inflammation index (SII), systemic inflammation response index (SIRI), neutrophil-to-lymphocyte ratio (NLR), and platelet-to-lymphocyte ratio (PLR), is strongly linked with poor overall and progression-free survival. On the contrary, a positive association was consistently fixed between a high lymphocyte-to-monocyte ratio (LMR) and a positive outcome, which highlights the prognostic power of host immune and inflammatory conditions in NPC. Our results are in line with prior evidence that systemic inflammation contributes to tumor proliferation, metastasis, and immune evasion. In the original article by Jiang et al. (19), who initially proposed the use of SII as a prognostic tool in NPC, the patients with high SII had significantly poorer overall survival, and SII had the strongest predictive value in comparison with the conventional ratios, like NLR and PLR (20). Similarly, my results also validated that high pre-treatment SIRI and SII were independent predictors of adverse outcomes during the intensity-modulated radiotherapy (IMRT) era. These findings reflect on our combined ratios of hazards, which show almost twice as many risks of mortality in patients with high levels of inflammatory markers.

A critical aspect that warrants further consideration is the role of EBV DNA, which is currently regarded as the most powerful prognostic biomarker in NPC. While EBV DNA reflects tumor burden and viral activity, inflammation-based indices such as SII and SIRI capture the systemic immune and inflammatory response of the host. These two biomarker categories may therefore provide complementary rather than overlapping information. Emerging evidence suggests that combining EBV DNA with systemic inflammatory markers can improve prognostic accuracy compared to either parameter alone. However, due to limited reporting in the included studies, we were unable to perform a stratified or combined analysis. Future prospective studies should investigate whether inflammation-based markers retain independent prognostic significance after adjustment for EBV DNA and whether integrated biomarker models can enhance risk stratification.

In addition to NPC, there is recent evidence of the prognostic applicability of inflammation-based indices in diverse malignancies (21). The research in esophageal squamous cell carcinoma and renal cell carcinoma has demonstrated that an increase in the levels of NLR, PLR, and SIRI correlates with poor survival rates (9, 22). It is important to note that (23) showed that baseline SII and LMR were both independent prognostic factors in advanced NPC immunotherapy patients, and there exists a dynamic association between tumor inflammation and immune modulation. Collectively, these investigations support the biological viability of our findings and the prognostic strength of systemic inflammation rates (24). SII and SIRI were always found to be the best predictors of poor prognosis in our analysis. These results align with previous research on colorectal and hepatocellular carcinoma in which an increase in SII was associated with recurrence and poor survival after curative surgery (25, 26). Such cross-cancer consistency indicates that increased neutrophil and platelet activity - major elements of SII and SIRI - could be indicative of a pro-tumor inflammatory microenvironment, which facilitates angiogenesis, metastasis, and therapy resistance.

Several previous meta-analyses have investigated the prognostic value of individual inflammatory markers in nasopharyngeal carcinoma. For instance (27), analyzed 4, 359 NPC patients and reported that an elevated neutrophil-to-lymphocyte ratio (NLR) was significantly associated with poorer overall survival (OS) (pooled HR ≈ 1.51–1.60). Similarly (28), confirmed the prognostic significance of NLR, demonstrating a significant association with reduced survival outcomes in NPC patients. Regarding platelet-to-lymphocyte ratio (PLR) (29), reported that high PLR was also associated with worse OS (pooled HR ≈ 1.30–1.50). In addition (30), demonstrated that both NLR and PLR were significantly correlated with adverse prognosis in NPC. More recently (31), reported that elevated inflammatory markers, including NLR and PLR, were associated with poorer survival, with pooled HRs for OS reaching approximately 1.78. However, these previous studies were largely limited to single or dual biomarkers, primarily focusing on NLR and PLR, with relatively smaller sample sizes and less comprehensive analyses. In contrast, the present study expands upon existing evidence by simultaneously evaluating multiple inflammation-based indices, including emerging markers such as the SII, SIRI, and PIV. This comprehensive approach not only confirms the prognostic value of traditional markers but also identifies SII and SIRI as potentially stronger predictors of survival, thereby providing a more integrated and updated understanding of inflammation-based prognostic biomarkers in NPC.

The pathophysiologic processes of such associations are multifactorial. High neutrophil and platelet counts mediate the progression of tumor development by secreting vascular endothelial growth factor (VEGF) and matrix metalloproteinases, which stimulate angiogenesis and extracellular matrix remodeling. Lymphopenia, on the other hand, is an indicator of defective cytotoxic immune response, diminishing tumor surveillance. Tumor-associated macrophage differentiation with the aid of monocytes further increases immune suppression and tumor invasiveness. These processes, combined, justify the high negative prognostic value of high SII, SIRI, NLR, and PLR values. Biomarkers based on inflammation have a number of clinical benefits. They are cheap, measurable regularly, and can be easily incorporated into the regular blood tests. These indices may be used in NPC management as an addition to traditional prognostic systems like the TNM staging system to represent the systemic inflammatory and immune environment that is missed by the traditional anatomic staging system. Notably, indices based on inflammation can also be used to stratify patients to increase the intensity of treatment or use in immunotherapy-based treatment, with the predictive value of SII and LMR indicating the same concept in immunotherapy-treated groups.

One important source of heterogeneity in this meta-analysis is the variability in cut-off values used to define high versus low levels of inflammatory markers across studies. For example, the threshold for NLR and PLR differed considerably depending on study design, patient population, and statistical methods such as receiver operating characteristic (ROC) analysis or median-based categorization. Such variability may influence the magnitude of hazard ratios and limit direct comparability between studies. Although subgroup analyses partially addressed this issue, residual heterogeneity may persist. This is consistent with previous meta-analyses of biomarkers, where differences in threshold definitions are a recognized contributor to between-study variability.

To improve the clinical applicability of inflammation-based biomarkers, future studies should aim to establish standardized cut-off values through large, multicenter prospective cohorts. The use of uniform statistical approaches and external validation is essential to ensure reproducibility. In addition, developing consensus guidelines or adopting continuous-variable models rather than dichotomized thresholds may further enhance the robustness and generalizability of these biomarkers in clinical practice.

This meta-analysis has a number of limitations. The main limitation is that the majority of included research was carried out in the Asian population, especially in China, where NPC is endemic. Even though this represents the natural epidemiology of NPC, it can restrict the external validity of our results to non-endemic areas. Clinically, geographic and ethnic NPC differences are important since disease subtype distribution, Epstein-Barr virus association, baseline inflammatory status, and treatment patterns may be dissimilar between populations. Thus, the predictive abilities and optimal levels of inflammation-related biomarkers that were found in endemic Asian cohort groups cannot necessarily be directly extrapolated to Western or other non-Asian groups. There is a need to have future multicenter prospective studies with wider geographic representation in order to validate these biomarkers and their applicability in different patient populations. In addition, cutoff points of inflammatory markers were inconsistent across studies, which were associated with a slight degree of heterogeneity. Normalization of cutoff values and future validation in mixed populations is required. Dynamic changes occurring in the treatment of inflammatory markers should also be studied in future research to determine their predictive power for therapeutic response and recurrence. Additionally, the lack of data on EBV DNA levels in the included studies prevented us from evaluating the interaction or combined prognostic value of inflammatory markers with this key biomarker. The variability in biomarker cut-off values across studies represents a potential limitation that may contribute to heterogeneity and affect the precision of pooled estimates.

## 5 Conclusion

This meta-analysis supports the fact that hematologic markers of inflammation, especially SII and SIRI, are effective and independent prognostic variables in nasopharyngeal carcinoma. Their common inclination to clinical practice can contribute to more personalized risk stratification and more customized methods of therapy. Prospective and translational studies are also justified by the future to explain their mechanism and streamline their use in NPC prognostic models.

## Data Availability

The original contributions presented in the study are included in the article/supplementary material. Further inquiries can be directed to the corresponding author.
