# Correction: Prognostic significance of inflammation-based markers in nasopharyngeal carcinoma: a systematic review and meta-analysis

**DOI:** 10.3389/fonc.2026.1965645

**Published:** 2026-08-21

**Authors:** Yajun Wang, Leyu Zhang, Qianyu Liu, Yi Wu

**Affiliations:** 1 Experiment Center for Medical Science Research, Kunming Medical University, Kunming, Yunnan, China; 2 School of Basic Medical Sciences, Kunming Medical University, Kunming, China; 3 School of Clinical Oncology, Kunming Medical University, Kunming, China

**Keywords:** meta-analysis, nasopharyngeal carcinoma, NLR, PLR, prognosis, SII, SIRI, systemic inflammation

There was a formatting issue in **Figure 1** as published. Some text elements in the original figure extended beyond the figure boundaries, which affected the proper visual presentation of the figure.

The corrected **Figure 1** appears below:

**Figure 1:**
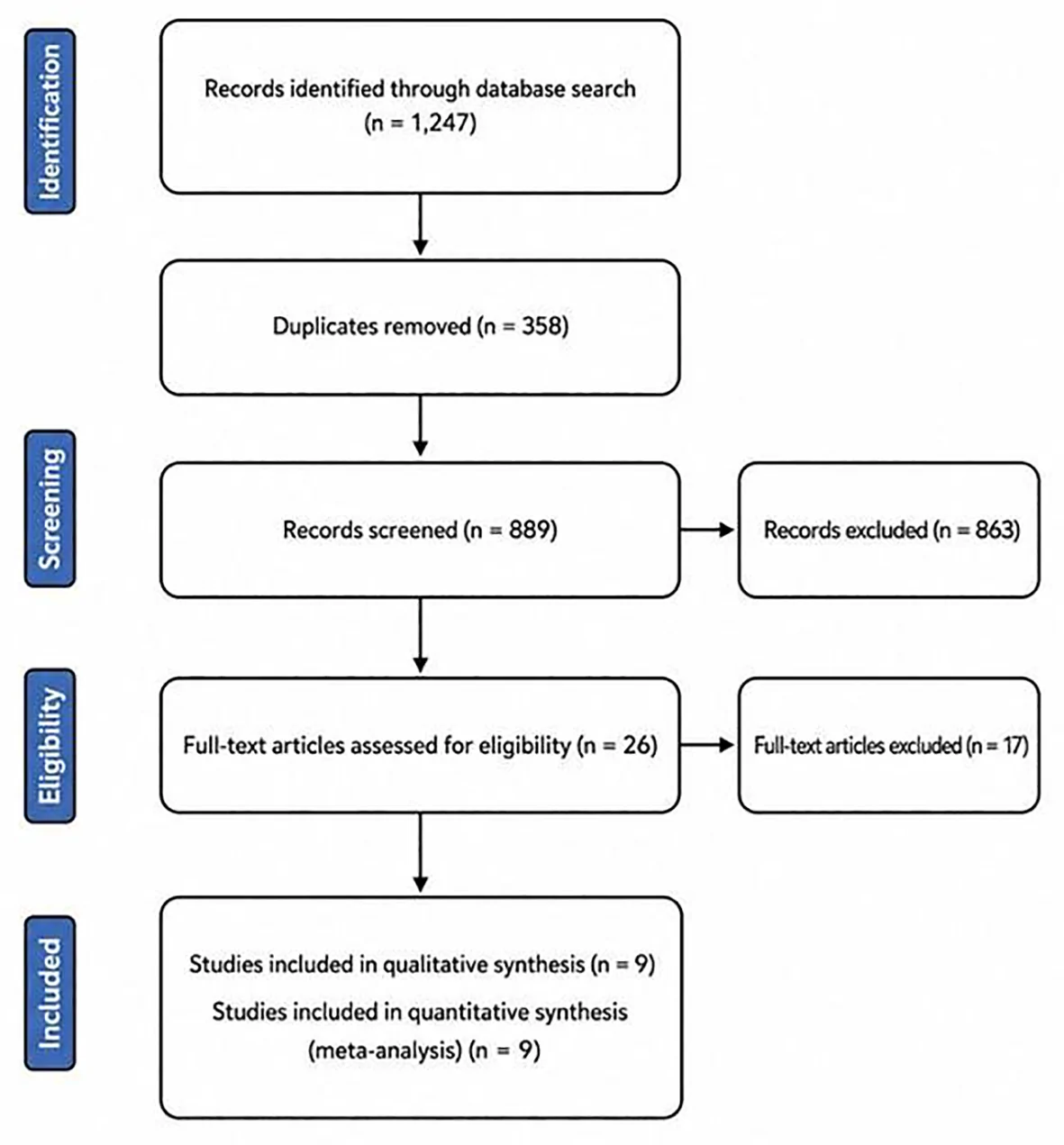
PRISMA flow diagram of the study selection process.

The original version of this article has been updated.

